# Differential Impact of Salinity Stress on Seeds Minerals, Storage Proteins, Fatty Acids, and Squalene Composition of New Quinoa Genotype, Grown in Hyper-Arid Desert Environments

**DOI:** 10.3389/fpls.2020.607102

**Published:** 2020-12-07

**Authors:** Kristina N. Toderich, Azimjon A. Mamadrahimov, Botir B. Khaitov, Aziz A. Karimov, Azamjon A. Soliev, Kameswara Rao Nanduri, Elena V. Shuyskaya

**Affiliations:** ^1^International Platform for Dryland Research and Education, Tottori University, Tottori, Japan; ^2^International Center for Biosaline Agriculture for Central Asia and Caucasus (ICBA-CAC), Tashkent, Uzbekistan; ^3^Institute of Bioorganic Chemistry Academy of Sciences of Uzbekistan, Tashkent, Uzbekistan; ^4^K.A. Timiryazev Institute of Plant Physiology Russian Academy of Sciences, Moscow, Russia

**Keywords:** *Chenopodium quinoa*, Amarantaceae, squalene, fatty acids, amino acids, proline, saline stress, desertification

## Abstract

The effects of climate change and soil salinization on dryland ecosystems are already widespread, and ensuring food security is a crucial challenge. In this article, we demonstrate changes in growth performance and seed quality of a new high-yielding quinoa genotype (Q5) exposed to sodium chloride (NaCl), sodium sulfate (Na_2_SO_4_), and mixed salts (NaCl + Na_2_SO_4_). Differential responses to salt stress in growth performance, seed yield, and seed quality were identified. High salinity (mixed Na_2_SO_4_ + NaCl) reduces plant height by ∼30%, shoot and root dry weights by ∼29%, head panicle length and panicle weight by 36–43%, and seed yield by 37%, compared with control conditions. However, the 1,000*-*seed weight changes insignificantly under salinity. High content of essential minerals, such as Fe, Zn, and Ca in quinoa Q5 seeds produced under salinity, gives the Q5 genotype a remarkable advantage for human consumption. Biomarkers detected in our studies show that the content of most essential amino acids is unchanged under salinity. The content of amino acids Pro, Gly, and Ile positively correlates with Na^+^ concentration in soil and seeds, whereas the content of squalene and most fatty acids negatively correlates. Variation in squalene content under increasing salinity is most likely due to toxic effects of sodium and chlorine ions as a result of the decrease in membrane permeability for ion movement as a protective reaction to an increase in the sodium ion concentration. Low squalene accumulation might also occur to redirect the NADPH cofactor to enhance the biosynthesis of proline in response to salinity, as both syntheses (squalene and proline) require NADPH. This evidence can potentially be used by the food and pharmaceutical industries in the development of new food and health products.

## Introduction

Rapidly increasing soil salinization in the world’s drylands is a major constraint in enhancing climate resilient ecosystem function and improving food security. The use of halophytes may be a viable solution for reducing pressure on freshwater resources and to utilize land affected by salinity (Toderich et al., 2018; Nikalje et al., 2019; Yamanaka and Toderich, 2020). Halophytes are salt-loving plants that possess an exceptional adaptation mechanism to abiotic stressors. *Arthrocnemum macrostachyum* (a coastal halophyte of Amaranthaceae) foliar extract application minimized the negative impacts of chloride salinity on soybean plants by increasing the proline, total free amino acids, total phenols, and ascorbic acid content (Osman et al., 2020).

Quinoa (*Chenopodium quinoa*), a facultative pseudocereal halophyte, has attracted worldwide interest because of its tolerance to harsh environments and superior nutritional value of seeds (Nowak et al., 2016; Nanduri et al., 2019). It, like other halophytes, can produce an economically valuable seed yield in a high range of salinities in which conventional crops cannot grow. Genetic enhancements of quinoa cultivars from its native Andes habitats were performed by different researchers in order to adapt this crop to different geographic conditions found in different dryland countries.

As a result, various cultivars of quinoa have been shown to exhibit different degrees of tolerance to abiotic and in particular saline stress (Peterson and Murphy, 2015; Hinojosa et al., 2018). Among the 121 germplasm accessions from the US Department of Agriculture (USDA), which were evaluated at the experimental station of the International Center for Biosaline Agriculture (ICBA), UAE, the quinoa Q5 improved genetic line has shown high plant growth performance, uniform early maturation, and stable high yield seed production. On-farm multilocation trial evaluations of quinoa germplasms under various climatic and soils salinity variables of Arabian Peninsula, Middle East North African, and Aral Sea Basin countries confirmed the exceptional adaptability of the (Q5) cultivar (Choukr-Allah et al., 2016).

There are more than 3,000 quinoa ecotypes whose potential and nutritional value have not been explored outside the Andes. Many studies have been performed to analyze the tolerance of different quinoa genotypes to abiotic stress in terms of its agronomic performance (Adolf et al., 2013; Ruiz et al., 2016; Mamedov et al., 2020), whereas a few have been conducted to explore the effects of high salinity response on the nutritional profile of quinoa seeds (Sampaio et al., 2020).

Information about the mechanisms involved in quinoa salinity tolerance is scarce and focuses on seed germination differences and physiological features response by comparing cultivars of different geographical origin (Shabala et al., 2013). Andean cultivars, for example, show high adaptability to NaCl stress due to proline production, the content of which was significantly higher than that of the control group seeds. Among the salts, Na_2_CO_3_ had the most detrimental effects on the germination of quinoa seeds, inhibiting the germination by ∼50%. Wilson et al. (2002) observed a significant reduction in plant height, when quinoa was grown in saline soil with a mixture of MgSO_4_, Na_2_SO_4_, Na_2_SO_4_, NaCl, and CaCl_2_ (3–19 dS m^–1^).

Intervarietal and intravarietal differences in seed mineral concentrations strongly suggest that both genotype and environmental factors were responsible for the mineral content variation among the quinoa cultivars along a geographical gradient. In conformity with studies of Iqbal et al. (2020) quinoa regulates excessive Na^+^ loads efficiently by sequestering it in leaf vacuoles and translocating it to older leaves. In another study, the mineral content of calcium (Ca), magnesium (Mg), zinc (Zn), and manganese (Mn) in quinoa seeds decreased in response to saline–sodic soil, e.g., in Larissa, Greece croplands (Karyotis et al., 2003). An X-ray microanalysis found high Na^+^ accumulation in the pericarp of quinoa seeds, but low amounts in the perisperm and embryo tissue (Sayed et al., 2017). According to Ali Hanan et al. (2017), concentrations of essential minerals such as Fe increase under high salinity conditions. The chemical composition of metabolites plays an important role in the osmotic adjustment to salinity stress in quinoa seeds (Aloisi et al., 2016). Seeds were also described as a good source of antioxidant compounds, although phenolic content and antioxidant and antimicrobial activities also varied among genotypes of quinoa (Park et al., 2017; Razzeto et al., 2019).

Recently, increased total polyphenolic content and antioxidant activity (AA) in methanol extracts of quinoa seeds harvested from plants grown under salinity have been observed, suggesting that stressful conditions may positively affect the seed’s content of these important bioactive compounds (Ruiz et al., 2016). Flour and the protein concentrate (PC) of seeds of amaranth, a close relative of quinoa, have been shown to contain polyphenols and to possess AA (Palombini et al., 2013; Aloisi et al., 2016).

Quinoa oil can also be used in cosmetics as a moisturizing agent of the skin due to a powerful combination of natural essential fatty acids (FAs) and vitamin E. This makes it a potent antioxidant/anti-inflammatory complex that helps restore barrier function of the skin epidermis and prevents premature signs of aging by enhancing collagen and elastin production. To date, these parameters have not been investigated in quinoa seeds, grown under arid and hyper-arid landscapes affected by inland salinization, which differs significantly from coastal salinization. In the last few decades, the incidence of abiotic stress has been accentuated by the increase in unpredictable weather patterns in the Central Asian landlocked lowlands areas. Investigation of quinoa response to chloride, sulfate, and mixed (chloride–sulfate) soil salinization that frequently occurs in this region can bring important information for more precise use, introduction, and outscaling of this climate-resilient superfood crop. There is concern that the chemical composition of local soils, which usually represents a mixture of salts, may alter the valuable nutritional profile of quinoa with a negative impact on the chemical compositions of quinoa seeds. Furthermore, abiotic stresses in these arid and hyper-arid areas habitually occur as combinations of two or more abiotic stress factors.

Quinoa varieties (e.g., CO407D, UDEC-1, Baer, and QQ 065) growing in Andes lowland environments exhibited extremely high tolerance to Na_2_SO_4_ and relatively high tolerance to NaCl in terms of agronomic performance, such as yield, plant height, and leaf greenness (Peterson and Murphy, 2015; Wu et al., 2016). However, seed nutritive quality under salinity stress remains to be evaluated when this crop is introduced in environments outside the Andes (Repo-Carrasco et al., 2003). We need to determine the underlying mechanisms of quinoa salinity tolerance and whether the content and quality of nutrients are influenced. The mechanisms causing differences in tolerance to chloride salts, sulfate salts, and mixed (chloride–sulfate) salts are still not well understood.

The current study aims to evaluate the seed quality profile of an improved genetic line of quinoa (Q5), introduced in salinized landlocked drylands, which radically differ from coastal salinized soil and Andes lowlands environments. Specific objectives were to investigate (i) quinoa plant performance under NaCl, Na_2_SO_4_ and the most frequently found mixed (NaCl + Na_2_SO_4_) salinity, which reflects the natural soil salinization in this region; (ii) mineral composition of the grain; (iii) amino acid profile of the grain; (iv) qualitative and quantitative analysis [chemical identification using total ion current (TIC) (thin-layer chromatography)] of FA methyl esters (FA-ME) composition, oil/fat proportion of non-saturated (NS) to saturated (ST) FA, and squalene content in a response to NaCl, Na_2_SO_4_, and mixed (NaCl + Na_2_SO_4_) types of salinity.

## Materials and Methods

### Experiment Area and Soil Analysis

The experiments were conducted during the 2017 and 2018 growing seasons, in the greenhouse of the Nukus branch of Agrarian University, in Karakalpakstan, Uzbekistan (42°28′N 59°36′E). For the present studies, three different types of soils representing the most common types of salinity found in the area were used.

These included chloride type soil (moderate level of salinization: 2.6 ± 0.21 Na^+^ mM/100 g soil), picked up from an abandoned rice field (N41°28′ E60°09′); sulfate-type soil (moderate level of salinization: 1.0 ± 0.09 Na^+^ mM/100 g soil), collected from a farmer’s field under forage crops (N42°36′ E 59°28′); and chloride–sulfate mixed soil (high level of salinization of 16.2 ± 2.03 Na^+^ mM/100 g soil), collected from the margins of a field with a strong saline crust (N42°36′ E59°28′) (Table 1). Non-saline soil collected from an overgrazed desert pasture (N 42°50′ E 60°00′) served as the control. The soils used in the experiments were obtained from a depth of 0–30 cm from representative fields. The soil in the region is characterized by a high proportion of fine sand and is moderately alkaline (pH 8.4) with a low humus content (0.6–1.08%) (Table 1). Humus content was determined by oxidation of soil organic matter with chromic acid to form carbon dioxide.

**TABLE 1:** Ion composition in the different type soils used in the experiment (mM/100 g soil).

Soil salinity type	Anions			Cations				Sum anions cations	Humus, %
	HCO3^–^	Cl^–^	SO_4_^2–^	Ca^2+^	Mg^2+^	Na^+^	K^+^		
Control	0.4 ± 0.04^b^	0.4 ± 0.03^c^	1.1 ± 0.09^b^	0.9 ± 0.11^b^	0.2 ± 0.03^c^	0.5 ± 0.07^*d*^	0.32 ± 0.02^a^	1.9/2.0	1.8
NaCl	0.5 ± 0.04^a^	2.0 ± 0.9^b^	1.4 ± 0.21^b^	0.7 ± 0.09^b^	0.4 ± 0.05^b^	2.6 ± 0.21^b^	0.11 ± 0.02^b^	3.9/3.8	0.9
Na_2_SO_4_	0.5 ± 0.05^a^	0.6 ± 0.04^c^	1.2 ± 0.13^b^	0.8 ± 0.07^b^	0.5 ± 0.06^b^	1.0 ± 0.09^c^	0.08 ± 0.01^b^	2.3/2.3	0.8
Na_2_SO_4_ + NaCl	0.2 ± 0.03^c^	16.7 ± 1.15^a^	14.5 ± 1.23^a^	6.2 ± 0.54^a^	8.9 ± 0.14^a^	16.2 ± 2.03^a^	0.10 ± 0.01^b^	31.3/31.5	0.6

*The values are means ± SEs. Different letters represent significant differences at the p < 0.05 level (Tukey pairwise comparison).*

Concentrations of main mineral ions were determined in water extracts of the seed mass (0.2 g dry mass/100 mL deionized water, shaken for 1 h and filtered). Sodium, potassium, and chloride ions were measured in the extracts using a pH/ion meter (“Expert-001.3”) in accordance with standard electrometric methods of measurement for ion-selective electrodes [Elit-031 (Kþ); Elit-261 (Cl_), Elis-112Na (Naþ)]. Sulfate–ion concentrations were measured in the same water extracts using UV/VIS Spectrophotometer V-530 (“Jasco”) by standard method with BaCl_2_ (Vorobyova and Makarenko, 2005).

### Plant Growth

The Q5 quinoa seeds used in these studies were provided by the ICBA, Regional Branch for Central Asia and Caucasus (ICBA-CAC). Five new salt- and drought-tolerant lines, named Q1–Q5, were developed by ICBA UAE through mass selections from accessions received from the USDA. Original seeds were from Bolivia (from where the collection was obtained by USDA), but they did not group with the other Bolivian lines when genotyped.

Instead, the Q1–Q5 lines grouped between Chilean lowland and Andean highland lines, so it is thought that these lines are probably a hybrid between the Andean highland and lowland types or an entirely new ecotype. Evaluation of these Q1–Q5 lines under marginal environments of Uzbekistan during 2015–2018 demonstrated a high performance of the Q5 line. Therefore, Q5 seeds were used in this study. Seeds were sown in 20-L plastic containers filled with 15 kg of soil collected from fields representing the four different soil types, including the control.

A total of six pots were used for each soil type, giving us six replications. Plants (one plant per pot) were grown under natural daylight conditions from April to end of September; the day temperature was maintained at 30°C, and night temperature at 22°C (16 h L/8 h D). Plants were also watered with 100–200 mL water supplemented with fertilizers (N:P:K 10:10:27; 0.4 g L^–1^) in the daytime. Seeds were collected at maturity starting from 91 days and up to 110 days, weighed, and stored in an airtight container at 4°C. Plant growth performance parameters—the height of the plant, shoot lengths, panicle weight, seed yield, and 1,000-seed weight—were then measured.

### Seed Chemical Analysis

#### Determination of Total Oil Content

Chemical analysis of quinoa seeds was carried out at the Institute of Bioorganic Chemistry, Tashkent. About 1 g of dry seed sample was crushed with pestle and mortar, and the homogenized mass was placed into an envelope through the filter article. The envelope with the sample was loaded into the Soxhlet extractor, and seed lipids were extracted with 50-mL boiling pure hexane (for gas chromatography MS SupraSolv < *cps*:*sup* > ®) by more than 20 repetitive Soxhlet cycles. Hexane-extracted seed samples were dried in a vacuum desiccator until constant weight, and oil content was calculated (in%) as loss of mass after Soxhlet processing (Wood et al., 1993).

#### Total Protein Determination

Protein content in the seeds was calculated as amino acids yield from acidic and alkaline hydrolysate’s chromatogram. Determination of amino acid content after both acidic and alkaline hydrolyses of defatted seed and free amino acids in deproteinated seed samples was carried out using reverse–high-performance liquid chromatography (RP-HPLC) [Agilent 1200, combined variable wavelength detector (VWD) and fluorescence detector (FLD)] after precolumn derivatization of the samples with *o*-phthalic aldehyde and phenylthiocyanate (Dai et al., 2014).

#### Free Amino Acid Content

Fifty milligrams of dry seeds was ground with 5 mL 40% methanol, containing 7.5% trichloroacetic acid (TCA) using mortar and pestle. The homogenate was centrifuged at 12,000 rpm for 3 min, and the deproteinizated solution containing amino acids was collected. TCA in solution was neutralized by concentrated NaOH (pH 8–10, controlled with indicator paper). Ten microliters of this deproteinizated extract was mixed with 190 μL of phenylisothiocyanate (PITC)–based derivatization reagent (5 μL PITC, 500 μL of 4 M Na-tetraborate, pH 10.5 and 495 μL of Acetonitrile). Derivatization in the form of phenylthiocarbamoil (PTC)–amino acids was completed by shaking at room temperature for 5 min. PTC derivatives were diluted by adding equal volume of 40% acetonitrile and loaded in HPLC column. Separation of PTC–amino acids was carried out using the HPLC equipment (VWD used as single detector at 280 nm) on RP column 0.46 × 25 cm XDB Zorbax C18.5 μm.

#### Total Amino Acid Content

Ten milligrams of defatted dry seed sample (protein content approximately 2 mg) was hydrolyzed with 2 mL of acidic mixture containing 6 M HCl (2 volumes), anhydrous trifluoroacetic acid (1 volume), and 0.5% 2-mercaptoethanol (2-ME). Hydrolysis was carried out at 170°C, 40 min under N_2_ in sealed ampules (Darbre, 1986). The hydrolysate was then evaporated to dryness using evaporator or SpeedVac system, suspended in 1 mL of distilled water, and centrifuged at 12,000 rpm, 2 min, to remove the insoluble contents. The amino acid content in acidic hydrolysate was determined by RP-HPLC using precolumn derivatization with *o*-phtalaldehyde (OPA) (Dorresteijn et al., 1996).

#### Determination of the Elementary Composition of Seeds

The seed samples were washed to remove surface contamination and dried in an oven at a temperature of 60°C. The samples were ground in a porcelain mortar to a homogenous state, and then two samples were weighed, one of 40 mg for analysis by short-lived radionuclides and the second of 90–100 mg to analyze medium- and long-lived radionuclides in labeled packets and packaged in plastic bags. The samples were then analyzed by neutron activation analysis method (De Soete et al., 1972). To determine the content of long-lived radionuclides, the samples were irradiated for 15 h. Samples were measured 1 month after irradiation by the relevant radionuclides. All measurements were performed on a germanium detector and spectrometer, connected to the PC. The determination of the elements of the different standards was used: interlaboratory, obtained by applying a known amount of the element on ash-less filter paper and the standard reference samples taken from IAEA Sabbage, IAEA Lichen, IAEA 359 and 336, and the comparator method (Parry, 1991; Abdurakhimov and Makhmudov, 2014).

#### Fatty Acid Composition of Quinoa Seed Oil

Anhydrous Na_2_SO_4_ was added to prepare the anhydrous hexane extract of FAs. The solvent and FA-ME were removed by evaporating, as described by Wirasnita et al. (2013). Fifty milligrams of oil was emulsified in 3 mL of alkaline methanol, containing 0.4 M KOH. The alkaline–methanol emulsion of oil was stirred using mechanical stirrer at 60–65°C for 10 min. After cooling with tap water, the reaction mixture was neutralized by adding an appropriate amount of conc. H_2_SO_4_ and then diluted with an equal volume of water. FA-ME was extracted from the reaction mixture three times with 1 mL of pure hexane for gas chromatography MS SupraSolv < *cps*:*sup* > ®. Hexane layers were combined and dehydrated with Na_2_SO_4_ by evaporation to dryness. Dry samples of FA-ME were dissolved in 1 mL of hexane prior to GC-MS analysis.

#### GC-MS Analysis of Fatty Acids

Two microliters of FA-ME sample was loaded to the GC-MS system in a split ratio of 1:20 to analyze the FA composition of the oil. Analyses were carried out using the TRACE 1310 TSQ 8000 (Thermo Fisher Scientific, United States) GC-MS system. The chromatographic conditions established were as follows: capillary column HP 5MS (30 m × 250 μm × 0.25 μm) and impregnated with a 5% biphenyl–dimethylsiloxane. The carrier gas was helium with a constant flow of 1.5 mL/min. The initial column temperature was set at 100°C with a delay of 1 min. Then the thermostat was heated to 180°C at a rate of 10°C/min, holding it at 230°C for 6 min, followed by a decrease in temperature to the initial state for 6 min at a rate of 40°C. The temperature of the injector and mass spectrometric detector was set at 250°C. The ionization method was performed by electron impact mode at 70 eV. Registration of chromatographic profile was generated after 3 min from the start to remove the signals of solvent. The chromatographic process was controlled by the XCalibur program in the interval of m/z 10–1,000 mass ranges. Identification of components was made using a combined reference library of mass spectrometric data of natural compounds “NIST” and Wiley. Total analysis time was 22.33 min. FA composition of quinoa seed oil samples was calculated from a TIC chromatograms of FAME Standard Calibration Mix C8:0–C24:0 (NuChek-Prep GLC 461C). TIC area of FA-ME peak was accepted as% content of the corresponding FAs. To calculate the quantity of squalene, we used an analytical standard of Squalene 111-02-4 Supelco.

### Statistical Analysis

Statistical analysis of the data was performed (analysis of variance with CropStat program), and means were compared using the Tukey test. Because differences among the various parameters in 2017 and 2018 were insignificant, the values were pooled before subjecting to the statistical analysis. Statistical software package R was used to perform a multivariate statistical approach using a principal component analysis (PCA) model.

## Results

### Plant Growth and Seeds Yield

Data analysis indicated a negative impact of soil salinity on plant growth and panicle weight (Table 2). High salinity content (mix of Na_2_SO_4_ + NaCl) reduces plant height by ∼30%, shoot and root dry weights by ∼29%, panicle height and panicle weight by 36–43% and seed output of each plant by 36.6% as compared to control group (Table 2). At the same time, the weight of 1,000 seeds has decreased only by a small amount (11.8%). The average salt content, both sulfate and chloride, had no effect on the accumulation of dry shoot biomass and panicle weight, but had a negative correlation with panicle height and seed output of the plant. Average chloride salinity significantly reduced plant height and root dry weight, in contrast to average sulfate salinity (Table 2).

**TABLE 2:** Quinoa growth parameters at maturity stage under different soil conditions.

Soil salinity type	Plant height, cm	Shoot dry weight, g	Root dry weight, g	Panicle height, cm	Panicle weight, g	Seed output of plant, g	Weight of 1,000 seeds, g
Control	132.7 ± 12.1^a^	192.3 ± 20.5^a^	81.3 ± 7.3^a^	42.4 ± 3.8^a^	63.0 ± 6.0^a^	42.6 ± 4.1^a^	1.7 ± 0.13^a^
NaCl	110.2 ± 9.3^b^	174.1 ± 15.7^a^	68.4 ± 5.1^b^	34.3 ± 3.2^b^	56.8 ± 5.4^a^	32.3 ± 3.0^b^	1.6 ± 0.15^a^
Na_2_SO_4_	119.8 ± 11.9^a b^	174.3 ± 16.1^a^	70.1 ± 6.9^a b^	35.7 ± 3.3^b^	52.6 ± 4.9^a^	34.3 ± 3.1^b^	1.6 ± 0.11^a^
Na_2_SO_4_ + NaCl	92.3 ± 7.4^c^	135.8 ± 14.2^b^	57.9 ± 4.1^c^	27.2 ± 2.6^c^	35.8 ± 3.2^b^	27.0 ± 1.9^c^	1.5 ± 0.14^a^

*The values are means ± SEs. Different letters represent significant differences at the p < 0.05 level (Tukey pairwise comparison).*

### Fatty Acids and Squalene Contents in Quinoa Seeds

**FIGURE 1:**
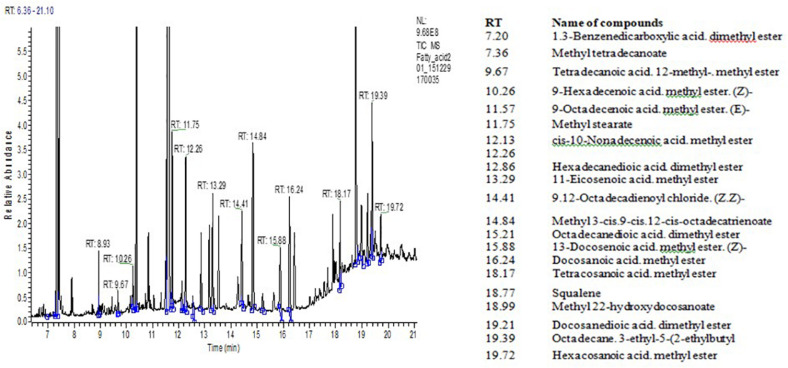
TIC chromatogram of fatty acids (FA) derived from Quinoa seed oil. Identification of FA was according to reference MS library analysis. Inserted table (right)—RT and the names of fatty acid methyl esters.

Data on FA-ME, oil/fat proportion of NS to ST FA, and squalene content in the seed oil of quinoa grown under different salinized conditions are shown in Table 3. The range of total lipid content in the seed samples varied between 4.95 and 6.42% per absolute dry mass of seeds. In quinoa grain, squalene content ranged between 0.148 and 0.256 g per 100 g of the seed mass. Figure 1 shows the TIC chromatogram of quinoa seed oil FA content. The squalene was detected at 18.77 min. Peak identification of FA composition was completed by using the Wiley reference mass spectra library and comparing retention indices of peaks before calculation of FA percentage content. Structural analysis by NIST library confirmed all the eluted peaks to be the substances of FAs and squalene. The probability of matching of the squalene peak with this substance was almost 90–95%. Values of oil/fat proportion of NS to ST FAs were considered the main criteria in evaluation of nutritive significance of quinoa grain. Oil of seeds extract of quinoa has favorable characteristics in terms of FA NS/ST proportion ratio that varies at 5.8–6.0. Quinoa seed oil is rich on polyunsaturated FA C_18__:__3_ (ω−3) and C_18__:__2_ (ω−6) content (Table 3). The palmitic acid (PA) **C_16__:__0_** value was higher in quinoa grown in under control conditions (11.54 ± 0.30%), compared to the samples from sodium chloride (9.46 ± 0.25%), sodium sulfate (8.77 ± 0.30%), and the high mixed salinity (8.49 ± 0.30%) (Table 3). Salinity had also affected the quantity of oleic acid (OA) **C_18__:__1_**. While the samples from the control group had 23.95 ± 0.70% per absolute seed dry mass, those from the sodium chloride, sodium sulfate, and the mixture of salts had 19.34 ± 0.60%, 18.81 ± 0.60%, and 16.81 ± 0.60% OA, respectively. Similarly, linoleic acid (LA) **C_18__:__2_** was also found to be higher in quinoa grown under normal conditions (53.98 ± 1.80%), and the concentration decreased to 43.01 ± 2.00%, 41.52 ± 2.00%, and 38.51 ± 1.70% under sodium chloride, sodium sulfate, and the mixed salinity, respectively (Table 3). Analysis of the data revealed differences in FA and squalene contents of the quinoa seeds under different soil conditions. High salinity (mixed Na_2_SO_4_ + NaCl) decreases nearly all studied FAs except stearic C_18__:__0_ and arachidic C_20__:__0_. Thus, the content of linolenic acid (C_18__:__3_) decreased by 53.4%, palmitoleic acid C_16__:__1_ by 35.7%, and other acids by 25–30% compared with the control conditions. The content of stearic acid C_18__:__0_ increased by ∼90%, and arachidic acid increased C_20__:__0_ by 11.4% (Table 3). Average salinity, both sulfate and chloride, did not affect the content of palmitoleic acid C16:1, but significantly reduced the content of oleic C_18__:__1_ and linoleic C_18__:__2_ acids. Medium sulfate salinity had a stronger effect on myristic C_14__:__0_, palmitic C_16__:__0_, stearic C_18__:__0_, and linolenic C_18__:__3_ acids, compared to medium chloride salinity. Conversely, chloride salinity has a stronger effect on arachidic acid C_20__:__0_ than sulfate salinity (Table 3). The squalene content decreased by 22% under conditions of strong (mixed Na_2_SO_4_ + NaCl) salinity, by 16% under conditions of medium sulfate salinity, and by 11.6% under conditions of medium chloride salinity (Table 3). Thus, the content of FAs and squalene is significantly influenced by the level of salinity, and the average sulfate salinity has a greater negative effect than chloride salinity.

**TABLE 3:** Fatty acids and squalene contents in quinoa seed oil from plants grown under different soil salinity (in g/100 g seed mass).

Fatty	LIT (10)	Soil salinity types			
		Control	NaCl	Na_2_SO_4_	NaCl + Na_2_SO_4_
Myristic C_14__:__0_	0.1 ± 2.4	0.47 ± 0.03^a^	0.42 ± 0.03^ab^	0.38 ± 0.03^b^	0.35 ± 0.04^b^
MyristoleicC_14__:__1_	0.1 ± 1.0	≤0.1^b^	0.15 ± 0.05^a^	≤0.1^b^	≤0.1^b^
Palmitic C_16__:__0_	9.2 ± 11.1	11.54 ± 0.30^a^	9.46 ± 0.25^b^	8.77 ± 0.30^c^	8.49 ± 0.30^c^
PalmitoleicC_16__:__1_	0.2 - 1.2	0.14 ± 0.01^a^	0.17 ± 0.02^a^	0.15 ± 0.02^a^	≤0.1^b^
Stearic C_18__:__0_	0.6 ± 1.1	0.92 ± 0.05^a^	0.95 ± 0.05^a^	0.71 ± 0.04^b^	1.71 ± 0.06^c^
Oleic C_18__:__1_	22.8 ± 29.5	23.95 ± 0.70^a^	19.34 ± 0.60^b^	18.81 ± 0.60^b^	16.81 ± 0.60^c^
Linoleic C_18__:__2_	48.1 ± 52.3	53.98 ± 1.80^a^	43.01 ± 2.00^b^	41.52 ± 2.00^b^	38.51 ± 1.70^c^
Linolenic C_18__:__3_	4.6 ± 8.0	8.62 ± 0.05^a^	5.30 ± 0.04^b^	4.72 ± 0.06^c^	4.02 ± 0.04^d^
Arachidic C_20__:__0_	ND*	0.35 ± 0.02^a^	0.43 ± 0.03^b^	0.40 ± 0.04^a^	0.39 ± 0.03^a^
NS/ST**		6.5	5.8	5.3	5.0
Squalene	ND	3.45 ± 0.02^a^	3.05 ± 0.03^b^	2.90 ± 0.04^c^	2.69 ± 0.03^d^

The values are means ± SEs. Different letters represent significant differences at the p < 0.05 level (Tukey pairwise comparison). *No data. **NS/ST, non-saturated (NS) to saturated (ST) FA.

**FIGURE 2:**
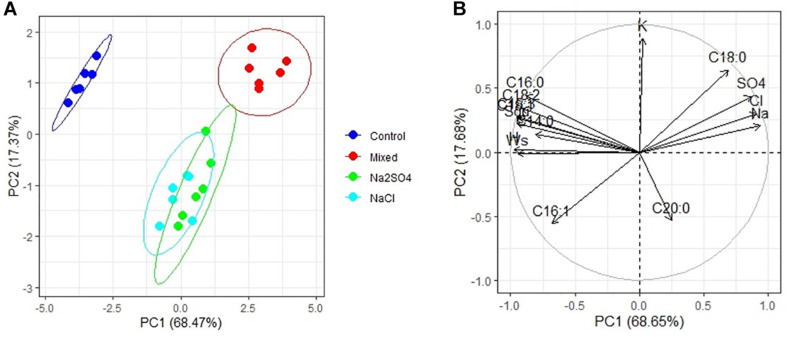
Principal component analysis (PCA) **(A)** score plot results comparing parameters of fatty acids and squalene (Squ) in quinoa seeds under different soil salinity and **(B)** multiple correlations of fatty acids and squalene in quinoa seeds, weight 1,000 seeds (Ws) and Na^+^, K^+^, Cl^–^, SO_4_^2–^, humus (H) contents in soil.

PCA has shown that there are three clear groups of FA in the seeds (Figure 2A): plants grown under controlled conditions (1), plants grown in either moderate sulfate or moderate chloride salinization (2), and plants grown in strong sulfate–chloride salinization (3). The main contribution to the division into three groups by the first main factor (PC1) is the content of C_18__:__1_ acid and squalene (Table 4). The second factor (PC2) separates plants grown at sulfate and chloride salinization from the control plants and plants grown at strong sulfate–chloride salinization. The main contribution to this division is the content of C_16__:__1_ and C_20__:__0_ acids (Table 4). Multiple correlation analyses also showed no principal difference between the FA content of quinoa seeds and the salinity chemistry (Figure 2B). At the same time, there was a strong positive correlation between the content of C_18__:__0_ and a negative correlation between the content of C_16__:__1_ in quinoa seeds and the content of sodium, chlorine, and sulfate ions in soil. There was also a negative correlation between the C_20__:__0_ acid content of quinoa seeds and the potassium ion content of soil. To varying degrees, negative correlations are observed between the content of other FAs and squalene in the seeds and the content of sodium, chlorine, and sulfate ions in the soil, as well as positive correlations with the weight of seeds and the content of humus in the soil.

**TABLE 4:** Factor loading of fatty acids (FA) and amino acids (AA) variables on axes 1 and 2 of the principal component analysis.

FA	P1	P2	AA	P1	P2
C14:0	−0.361	0.129	Asx	0.27584	−0.2529
C16:0	−0.379	−0.193	Glx	0.09916	−0.1804
C16:1	−0.251	0.577	Ser	0.28269	0.18125
C18:0	0.215	−0.441	HIS	0.20599	−0.2524
C18:1	−0.393	−0.087	THR	0.15491	−0.2467
C18:2	−0.385	−0.129	UNK/Met	0.19645	0.2698
C18:3	−0.388	−0.112	Gly	**0.29309**	0.17982
C20:0	0.100	0.615	ARG	0.27283	−0.0731
Squ	−0.392	−0.062	Tyr	0.22704	**0.3124**
			Cys	**0.28287**	−0.0019
			Ala	**0.28775**	0.18406
			TRP	0.23725	−0.2243
			VAL/MET	0.15452	−0.2182
			PHE	0.174	−0.1771
			ILE	0.27396	0.2324
			LEU	0.23391	0.07064
			LYS	0.07773	**−0.3848**
			Pro	0.20496	0.2899
			Total AA	0.25727	−0.2893

*Bold types indicate the highest values.*

### Amino Acid Composition of Quinoa Seeds

Soil salinity, regardless of degree and chemistry, did not affect the content of amino acids Arg, Asx, Glx, His, Phe, Trp, and Val/Met, but significantly increased the content of Gly (by 6–23%), Tyr (by 27–38%), and Pro (12–48%) in quinoa seeds compared to control conditions. Under strong salinity (mixed Na_2_SO_4_ + NaCl), the Lys content decreased by 22%, and the Gly, Tyr, Ala, and Ile in quinoa seeds increased by 19–60%, as compared to the control conditions (Table 5). Under conditions of moderate sulfate salinity, a significant increase in the content of Thr (by 12–17%), Ser (by 26%), and Cys (by 61%) was observed, while under conditions of moderate chloride salinity, a significant increase in the content of Leu (by 32%) was observed in quinoa seeds vs. control conditions.

**TABLE 5:** Amino acid contents in quinoa seeds produced under different soil salinity (in g/100 g seed mass).

RT	AA	Soil salinity types			
		Control	NaCl	Na_2_SO_4_	NaCl + Na_2_SO_4_
2.87	Asx	10.99 ± 1.20^a^	11.32 ± 1.30^a^	10.64 ± 1.20^a^	10.14 ± 1.00^a^
4.05	Glx	12.66 ± 1.25^a^	12.96 ± 0.50^a^	13.12 ± 1.10^a^	11.44 ± 1.50^a^
7.16	Ser	4.61 ± 0.35^b^	5.62 ± 0.50^a b^	5.79 ± 0.40^a^	5.53 ± 0.50^a b^
7.34	**HIS**	**4.90 ±** 0.35^a^	**4.75 ±** 0.30^a^	**5.18 ±** 0.50^a^	**4.40 ±** 0.60**^a^**
8.25	**THR**	**5.38 ±** 0.50^a^	**4.47 ±** 0.40^b^	**4.72 ±** 0.40^a^	**4.96 ±** 0.40^a^
8.51	UNK/Met→O	3.34 ± 0.20^a^	3.44 ± 0.50^a^	4.01 ± 0.30^a^	4.88 ± 0.30^b^
8.62	Gly	3.96 ± 0.20^a^	4.60 ± 0.50^b^	4.21 ± 0.40^b^	4.86 ± 0.30^b^
9.57	**ARG**	**8.04 ±** 0.75^a^	**8.89 ±** 0.40^a^	**8.48 ±** 0.40^a^	**8.08 ±** 0.40^a^
9.96	Tyr	3.59 ± 0.20^a^	4.78 ± 0.20^b^	4.57 ± 0.20^b^	4.94 ± 0.35^b^
10.97	Cys	1.13 ± 0.15^a^	1.40 ± 0.45^a b^	1.82 ± 0.45^b^	1.35 ± 0.45^a b^
12.41	Ala	3.79 ± 0.20^a^	4.14 ± 0.30^a b^	3.95 ± 0.20^a b^	4.51 ± 0.40^b^
12.85	TRP*	**0.57 ±** 0.05^a^	**0.47 ±** 0.25^a^	**0.51 ±** 0.45^a^	**0.49 ±** 0.35^a^
13.85	**VAL**/**MET**	**8.45 ±** 0.75^a^	**7.91 ±** 0.60^a^	**7.15 ±** 0.40^a^	**8.05 ±** 0.20^a^
14.16	**PHE**	**5.86 ±** 0.60^a^	**4.99 ±** 0.80^a^	**4.93 ±** 0.30^a^	**5.62 ±** 0.40^a^
15.02	**ILE**	**2.16 ±** 0.20^a^	**3.12 ±** 0.40^b^	**2.65 ±** 0.50^a b^	**3.44 ±** 0.40^b^
15.57	**LEU**	**3.16 ±** 0.20^a^	**4.17 ±** 0.20^b^	**3.32 ±** 0.20^a^	**3.57 ±** 0.30^a^
16.96	**LYS**	**13.22 ±** 1.35^a^	**12.99 ±** 0.30^a^	**11.44 ±** 1.00^a b^	**10.34 ±** 0.40^b^
18.47	Pro**	2.16 ± 0.25^a^	2.42 ± 0.35^a b^	2.98 ± 0.15^b^	3.19 ± 0.15^b^
**Total AA = protein%**		14.4 ± 1.3^a^	14.2 ± 1.1^a^	13.9 ± 1.3^a^	13.2 ± 1.5^a^

The values are means ± SEs. Different letters represent significant differences at the p < 0.05 level (Tukey pairwise comparison). *Trp calculated from alkaline hydrolysate. **Proderivatization with FMOC. Bold and uppercase types indicate essential amino acid (for human; His and Arg for children). Quantity of amino acids indicated as g/100 g of protein; protein content calculated as total yield of amino acids (g) from 100 g of seed mass. Position with RT 8.51 min unidentified. Similar RT with methionine sulfone. AA, amino acid; Asx, asparagine and aspartic acid; Glx, glutamate and glutamine; Ser, serine; HIS, histidine; THR, tireonine; UNK/Met, methionine; Gly, glutamic acid; ARG, arginine; Tyr,tyrosine; Cys, cysteine and cystin; Ala, alanine; TRP, tryptophan; VAL/MET, valine/methionine; PHE, phenylalanine; ILE, isoleucine; LEU, leucine; LYS, lysine; Pro, proline.

**FIGURE 3:**
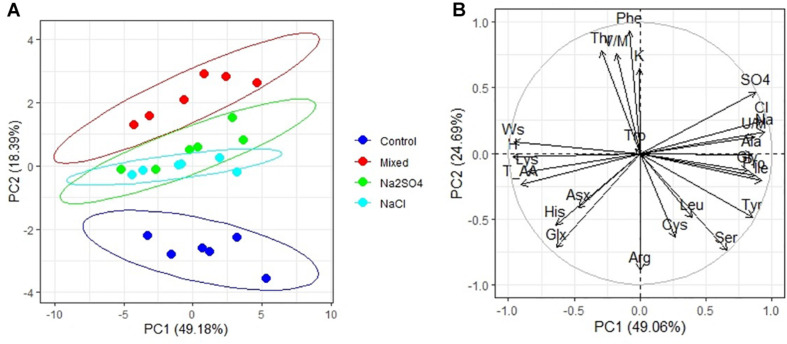
Principal component analysis (PCA) **(A)** score plot results comparing parameters of amino acids in quinoa seeds under different soil salinity and **(B)** multiple correlations of amino acids in quinoa seeds, weight 1,000 seeds (Ws) and Na^+^, K^+^, Cl^–^, SO_4_^2–^, humus (H) contents in soil.

PCA has shown that there are three clear groups of amino acid contents (Figure 3A): from plants grown under control conditions (1), from plants grown in both sulfate and chloride salinization (2), and from plants grown under strong sulfate–chloride salinization (3). The main difference between the three groups is the Tyr and Lys content (PC2) (Table 4). Multiple correlation analysis also showed that there was no difference between the amino acid content and the salinity chemistry of quinoa seeds (Figure 3B). In doing so, a positive relationship was found between Ala, Gly, Pro, Ile, and Unk and a negative relationship between Glx, His, Asx, and Lys content in quinoa seeds and the content of sodium, chloride, and sulfate ions in soil (Figure 3B). Despite the negative correlation between total amino acids in quinoa seeds and the content of sodium, chloride, and sulfate ions in soil (Figure 3B), no significant differences were found between the content of total amino acids under salinity and under control conditions (Table 5).

### Element Content in Quinoa Seeds

**FIGURE 4:**
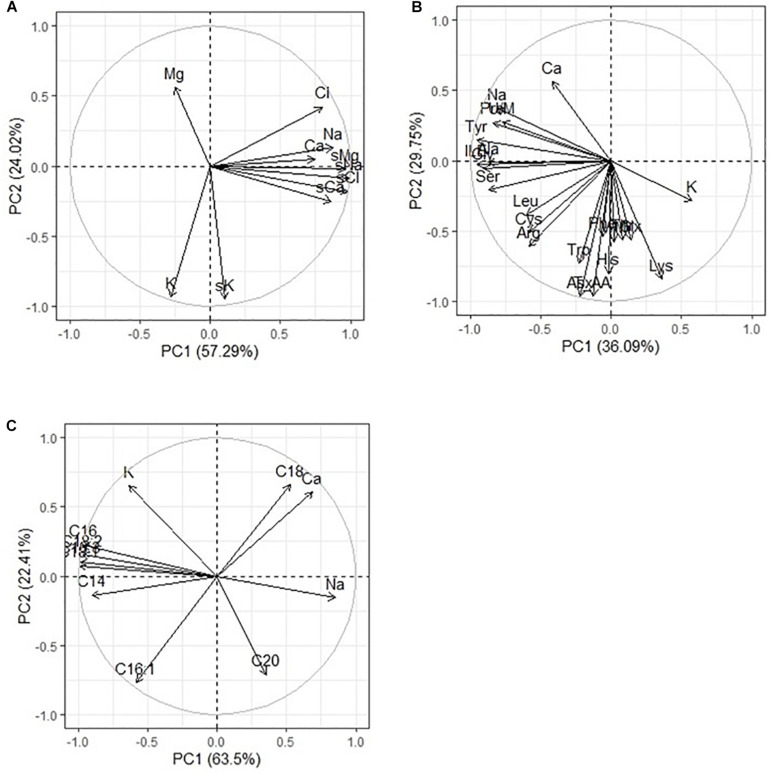
Principal component analysis (PCA) of multiple correlations Na. K, Ca, Mg, and Cl contents in soils and in quinoa seeds **(A)**; Na, K, Ca contents in quinoa seeds and **(B)** amino acids and **(C)** fatty acids in quinoa seeds under different soil salinity.

Analysis of the elemental composition of quinoa seeds under conditions of different chemistry and salinity showed an increase in the content of Na (by 13–31%), Cl (by 90–133%), Fe (by 3–77%), and Zn (by 8–33%) compared to control (Table 6). At the same time, the content of K (by 12–23%), Cu (by 4–44%), and Sb (by 84–87%) decreased. With strong salinity (mixed Na_2_SO_4_ + NaCl), a decrease in the content of Br (by 81%) and Co (by 52%) was observed, and an increase in the content of Ca (by 92%), Cr (by 33%), and Sc (by 44%). With a moderate salinity, both sulfate and chloride, a twofold increase in the Ba content in quinoa seeds was observed in comparison with the control conditions (Table 6). Na, K, Ca, and Cl contents in quinoa seeds positively correlated with Na^+^, K^+^, Ca^2+^, and C^–^ contents in soils, respectively. However, the Mg content in quinoa seeds did not correlate with its content in soils (Figure 4A).

**TABLE 6:** Mineral content (μ/g) in quinoa seeds produced under different soil salinity.

Element	Soil salinity types			
	Control	NaCl	Na_2_SO_4_	NaCl + Na_2_SO_4_
Mg	2,300	2,700	1,900	2,200
Cl	1,160	2,200	2,540	2,710
Mn	19	19	15	30
Cu	68	63	65	38
Na	187	212	238	245
K	13,200	10,210	11,500	11,600
Mo	<0.1	0.32	<0.1	0.49
Lu	<0.001	<0.001	<0.001	<0.001
U	<0.01	<0.01	<0.01	<0.01
Yb	<0.001	<0.001	<0.001	<0.001
Au	0.0017	0.0015	0.0013	0.0017
Nd	<1.0	<1.0	<1.0	<1.0
As	<0.1	<0.1	<0.1	<0.1
Br	4.2	4.6	2.2	0.80
Ca	780	930	620	1,500
La	<0.01	0.020	<0.01	0.055
Ce	<0.1	<0.1	<0.1	<0.1
Se	<0.1	<0.1	<0.1	0.12
Hg	<0.01	0.015	0.017	<0.01
Cr	0.36	0.36	0.44	0.48
Ba	<10	34	36	2.9
Sr	16	36	15	15
Sc	0.0090	0.0088	0.0069	0.013
Rb	5.9	8.1	5.9	5.8
Zn	36	39	39	48
Co	0.12	0.12	0.094	0.058
Fe	62	70	64	110
Sb	0.17	0.022	0.027	0.024

## Discussion

Although pseudocereals such as quinoa (*C. quinoa*) are valuable sources of nutrients, they are relatively understudied in terms of agronomic performance and elementary compounds composition response to harsh arid and hyper-arid landlocked saline environments. The strongly genotype-dependent responses to salinity confirm that quinoa is a rich source of genetic variation with respect to stress tolerance and that they are useful for further improving adaptation of this species to diverse environments (Nanduri et al., 2019). The pot experiment (Abdelaziz and Redouane, 2020) has shown that heat and water stress affected all phenotypic and physiological traits in Q1–Q5. Our current and previous findings (Mamadrahimov et al., 2016; Nanduri et al., 2019) show that, in some cases, abiotic stresses, e.g., soil salinity along a geographical gradient, induce changes in the nutritional properties of quinoa grains. Quinoa Q_5_ improved genetic line and not only endured salinity but produced good-quality seeds under saline conditions to a higher or similar degree to quinoa from the Andean native habitats.

Regarding the effects of Na_2_SO_4_ stress, there are studies showing that for many plants there is a trend of greater sulfate tolerance than chloride tolerance (Wilson et al., 2002; Peterson et al., 2015). Such tolerance was not confirmed by our results. Quinoa Q_5_ cultivar shows that the germination rate at 100 and 200 mM (Na_2_SO_4_) was, respectively, 10 and 25% lower, compared with the germination rate at the same concentrations of NaCl. Medium sulfate salinity in Q_5_, as was determined in this study, has more impact on the FA content of seeds than sodium chloride. Also, as in the case of NaCl, the germination of Q_5_ line seeds in the presence of Na_2_SO_4_, regardless of the concentration used, was significantly affected compared to the untreated seeds.

Choukr-Allah et al. (2016) reported a detrimental effect of water salinity (2.3, 16.3, and 18.9 dSm^–1^) on the chemical composition of quinoa seeds, particularly in the mineral content of the seeds. The results of this experiment also showed a high degree of variability in the performance of the nutritional profiles of quinoa seeds under various salinity stress. We have detected high sodium (Na) content in the quinoa seeds harvested from saline conditions as described earlier by Choukr-Allah et al. (2016). Quinoa seeds are recognized as a nutritious food source, owing to their high protein content with all essential amino acids, lack of gluten, and richness in several minerals such as Ca, Mg, Cu, Mn, P, Fe, and others (Peterson and Murphy, 2015). More importantly, even under the saline growing conditions, the nutritional value of quinoa seeds, especially the high protein content and all essential amino acids, high mineral content (e.g., Ca, Mg, Fe), and health-promoting compounds, such as flavonoids, remained mostly unaltered (Vega-Gálvez et al., 2010). In our experiments, no significant changes were observed in the content of Mg, Mn, Cu, Co, Ca, Se, Zn, and Fe under moderate chloride and sulfate soil salinity, when compared with those under control conditions (Table 6). The increases in Mn (by 58%), Fe (by 77.4%), and Ca (by 92%) contents in seed extracts from the strong mixed soil salinity do not support the earlier findings of Karyotis et al. (2003), who found a decrease in the mineral content of Ca, Mg, Zn, and Mn in quinoa seeds in response to saline-sodic soils. Under strong mixed salinity of NaCl and Na_2_SO_4_, a significant 1.3- to 2.3-fold accumulation of Cl and Na in quinoa seeds is also observed (Table 6 and Figure 4A). And at a given salinity, the highest level of reduction of quinoa nutritional compounds was detected. This might be due to the response of the plant protective system, or the mixed saline type of soil probably has more of these microelements.

An overview of literature has shown that the protein quantity and quality of quinoa are generally superior to those of cereal grains, while offering gluten-free property and high digestibility. Quinoa grain contains a balanced composition of essential amino acids with a high proportion of lysine, an amino acid deficient in cereal crops (FAO, 2013; U.S. Department of Agriculture, 2018). Protein/amino acid content are essential bioactive compounds of the food value of plant seeds. Performance of amino acid, FA contents, and nutritional quality of quinoa seeds under extreme growing environment will give a clear idea of how to advance this crop in marginal conditions. The major components of amino acids in whole seeds are included in storage protein/peptide and other proteinaceous constituents. However, some minor amounts can also be found as free amino acids. In this study, we determined total amino acid content after acidic/alkaline hydrolysis of defatted seed and free amino acids in deproteinized seed samples. Comparing the amino acids profiles of seeds under control and salinity showed that even strong mixed Na_2_SO_4_ + NaCl soil salinity did not affect the content of many essential amino acids (Arg, Asx, Glx, His, Phe, Trp, Val/Met) and increase in content of Gly, Tyr, Pro, Gly, Tyr, Ala, and Ile (Table 5 and Figure 3B), which is confirmed by significant correlations with the sodium content in seeds (Figure 4B). Of all the studied amino acids, only the content of Lys was decreased under heavy salinity (mixed Na_2_SO_4_ + NaCl). Overall, results show that salinity does not lower the seed amino acid content.

The accumulation of organic osmolytes, such as proline (a multifunctional amino acids), from the late embryogenesis plays a key role in maintaining the low intracellular osmotic potential of plants and in preventing the harmful effects of salinity stress on embryo development (Wang et al., 2015). Proline content, as described (in *Amaranthus mangostanus*) by Bhargava and Srivastava (2020), was significantly increased at high salt concentrations (90–120 mmol L_1). Some of authors indicated that the capacity to accumulate more storage proteins in quinoa seeds plays an important role in the initial stages of seed germination and seedlings establishment, one of the most sensitive to salinity stress seed ontogenetic development stages (Koyro and Eisa, 2008). Increase in proline may influence seed viability (Sano et al., 2016) and can ultimately lead to the production of protein to be used in the food industry and as dietary complements for their high protein level, functional properties, and low content of antinutritional factors (Escudero et al., 2011). By taking into consideration that quinoa, like other closely related chenopods, is characterized by a deep physiological seed dormancy, i.e., has short-term seed viability, we assumed that extending the seed dormancy period might play a crucial role in seed breeding and seed production programs. Proline accumulation was found to be dependent on the species and the plant organs (Verbruggen and Hermans, 2008; Bhargava and Srivastava, 2020); moreover, the genotypic differences in the proline concentration have been previously reported in sunflower (Canavar et al., 2014). As it was described by Abbas et al. (2014), the highly accumulated proline genotypes were more tolerant to abiotic stress. Proline accumulation in response to stress could be utilized in biosynthesis of proline-rich proteins. These proteins protect the plants from various stresses and help plants to recover from stress more rapidly (Hayat et al., 2012).

Quinoa (*C. quinoa* Willd.) seeds store about 12–15% of proteins with high biological value (Capraro et al., 2020). Our current studies show that soil salinity affected the amino acid content in quinoa seeds to a much lesser extent than the FA content. The content of most essential amino acids for human consumption and child development in quinoa seeds did not change under salinity conditions. Moreover, increases in content of major Fe, Mn, and Ca in combination with an increase in ST FAs, stearic acids, and amino acids (Ala. Pro., Gly, ILE, LEU) under mixed salinity (NaCl + Na_2_SO_4_) in quinoa (e.g., Q5) seed extract confirm the outstanding nutritional value of seeds with respect to salinity stress tolerance Further investigation of new quinoa germplasm will open avenues for improving adaptation of this species to diverse harsh environments. The accumulation of proline is often observed in response to salt stress, and it is most pronounced in roots (Hmidi et al., 2018). In this case, proline can perform a stress-protective function from the toxic effects of salts. Proline can destabilize double-helix DNA by lowering its melting point and increasing sensitivity to SI nuclease (Rajendrakumar et al., 1997). Under saline conditions, osmolyte destabilization of DNA has important cellular effects. Salts that accumulate during salt stress can also overstabilize the double helix, which can adversely inhibit DNA function in replication and transcription (Scott et al., 2019). Proline can also be a substitute energy source if necessary; proline degrades and releases energy as a reducing agent (Polavarapu et al., 2014).

Peiretti et al. (2013), in their studies revealed that the chemical composition of quinoa is closely connected to development of the plant with the quality of crop decreasing with increasing morphological stages. The pattern of FAs in the seed was characterized by PA (C_16__:__0_), OA (C_18__:__1__*n*__–__9_), and LA (C_18__:__2__*n*__–__6_). Among main FAs of the plant during growth, α-linolenic acid (ALA, C_18__:__3__*n*__–__3_) was the most abundant FA. The increase in ALA in plant seed content plays an important role in regulating intracellular FA unsaturation to resist salt stress (Upchurch, 2008). Syntheses of oil and FAs of chenopods were largely influenced by salinity effect and pronounced during seed maturity developmental stage that induced the declining of seed yield value (Elferjani and Soolanayakanahally, 2018). Multiple correlation analysis in our experiment showed no principal difference between the FA content of quinoa seeds and the salinity chemistry (Figure 2B). The value of OA pattern in quinoa seeds was 22.8 ± 29.5%, which is higher than described early by other authors (Altuna et al., 2018). Interestingly, the values of LA from the control were found to be higher than those reported in literature (48.1 ± 52.3%, respectively). Arachidic acid appears to be largely associated with climatic conditions, and the oil content, oil quality, and seed yield of canola significantly increase with increasing organic matter content of soil during seed development. At the same time, there was a positive correlation between the content of C_18__:__0_ and a negative correlation between the content of C_16__:__1_ in quinoa seeds and the content of sodium, chlorine, and sulfate ions in soil. However, a comparative analysis of the content of these FAs and the Na, K, and Ca accumulation in seeds showed a greater dependence of the content of these FAs on Ca than on sodium content (Figure 4C). Perhaps this is due to the role of these FAs in the activation of embryo Ca^2+^ -regulated protein kinase at different Ca concentrations (Lucantoni and Polya, 1987). There was also a negative correlation C_20__:__0_ content of quinoa seeds with the potassium ion content of soil (Figure 2B), as well as with K content in seeds (Figure 4C). To varying degrees, negative correlations are observed between the content of other FAs and squalene in the seeds and the content of sodium, chlorine, and sulfate ions in the soil, and this was confirmed by the same negative correlation with Na content in seeds (Figure 4C).

**FIGURE 5:**
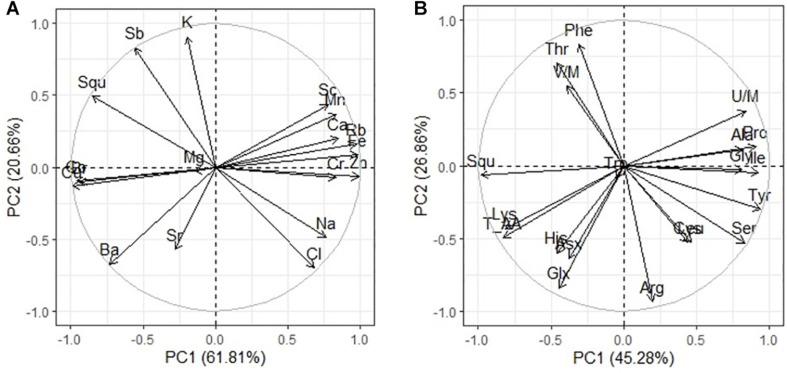
Principal component analysis (PCA) of multiple correlations squalene and elements contents in quinoa seeds **(A)**; squalene and amino acids in quinoa seeds under different soil salinity **(B)**.

The results also show that the seed yield (weight of 1,000 seeds), oil content, and oil quality of Quinoa positively correlate with humus content in soil. This study revealed that the nutrient matter content of poor saline soils should be ameliorated not only to obtain higher crop yields but also to improve the quality of production. The results of this study will be useful for agronomic and genetic research in determining the optimal harvest period. The significance of the results lies in the demonstration of the feasibility of seed growth stages at which developing quinoa seed could be modified to change oil and FA contents. A similar conclusion was made by Fadul (2015), where increase in arachidic acids appears to be largely associated with climatic conditions, and the oil content, oil quality, and seed yield of canola significantly increase with increasing organic matter content of soil during seed development. Results of this study showed that quinoa seeds are rich on polyunsaturated FA C_18__:__3_ (ω-3) and C_18__:__2_ (ω-6) and contain a considerable amount of squalene, which make them invaluable nutritional food for daily consumption. Incidentally, this is the first detailed report on the occurrence of squalene in the seed of quinoa, exposed to high soil salinity chemistry. The content of squalene in Quinoa (e.g., Q_5_) grains detected by using ion chromatography (TIC) varied from 2.69 to 3.45 g/100 g seed mass. Our results demonstrated that the total amount of squalene was changed in response to salinity stress. There was negative correlation between the content of squalene and the content of sodium, chlorine, and sulfate ion in the soil (Figure 2B) and a strong negative correlation with the accumulation of sodium and chlorine in the seeds (Figure 5A). A literature overview shows that plans containing squalene are olive oil (564 mg/100 g), soybean oil (9.9 mg/100 g), grape oil (19.1 mg/100 g), and *Amaranthus* (5,942 mg/100 g). However, of these species, olive seeds is used only for extracting commercial squalene despite the highest content is reported for *Amaranthus* (He et al., 2002). Several authors pointed out that squalene content in amaranth could be affected by environmental conditions, such as drought and water availability (He et al., 2002; Bozorov et al., 2018). Quinoa like *Amaranthus*, buckwheat, are important grains that provide a high quality of lipids and have health-promoting compounds. Squalene has a unique ability to saturate cells with oxygen. Allegedly, this is because squalene as an NS compound is unstable. However, the significance of squalene for the plant metabolism is not clear yet and requires further experimental studies. Like many other terpenoids, squalene is soluble in fats. Antitumoral and antiaging effects of squalene make it one of the powerful biological substances (Hill and Connolly, 2015). These physiological effects are generally well-tolerated and safe. The mechanisms of antitumor effects of squalene studied so far include decreasing farnesyl pyrophosphate (FPP) and free radical levels in cells, inhibition via negative feedback to the HMG-CoA enzyme, lessening the synthesis of FPP (Newmark, 1999; Reddy and Couvreur, 2009). The rich squalene content in quinoa seeds supports the levels and patterns of seed development and maturation stages due to its extraordinary antioxidant properties (Peiretti et al., 2013). Thus, the results of this study showed that the content of FAs and squalene in quinoa seeds is more influenced by the level of salinity than by chemistry, but moderate sulfate salinity has a greater negative effect than chloride salinity. Strong salinity (mixed Na_2_SO_4_ + NaCl) resulted in an increase in the content of two FAs (C_18__:__0_ and C_20__:__0_) and a decrease in the content of the remaining FAs and squalene.

**FIGURE 6:**
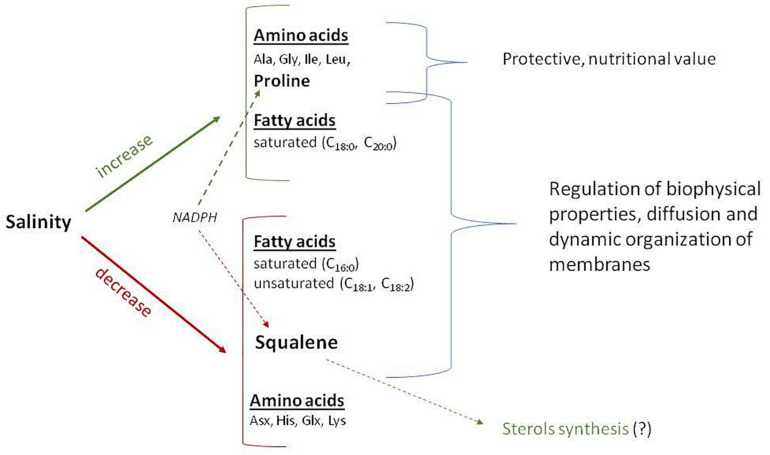
Schematic presentation of the pathway for certain important seed chemical compounds as affected by salinity in *Chenopodium quinoa* Willd (e.g., Q5 genotype). The red represents decreased, whereas the green represents increased.

The negative correlation of squalene content with the content of amino acids Pro, Gly, and Ile (Figure 5B) may be secondary, as the contents of amino acids Pro, Gly, and Ile positively correlate with the level of soil salinity (Figure 3B). Negative effects of salinization on plants may be due to low osmotic potential of the soil solution (osmotic stress) or toxic ionic effects (ionic stress) or a combination of these factors. Decrease in squalene content when salinity is increased is most likely due to toxic effects of sodium and chlorine ions. Squalene, an isoprenoid, is an important precursor for the biosynthesis of sterols, steroids, and ubiquinones. However, only 10% of the total quantity of squalene in the membrane is metabolically active for sterol synthesis, whereas the rest of it is stored, together with triacylglycerides and complex sterol esters as lipid droplets (Spanova et al., 2012). Because of its unpolarized nature, squalene is found in the hydrophobix center of the lipid bilayer, and it increases the hardness and size of the cell membrane when it assumes a hexagonal shape. Research of the lipid–protein interactions in cell membranes showed that squalene increases the polarity and hydrophobic interactions; it aids in regeneration of cell membranes, functional regulation of proteins, and ion transport (Orsini et al., 2011). The key cell membrane components are NS lipids, which, together with squalene, can regulate biophysical properties, passive diffusion, and dynamic organization of the membranes. Membrane viscosity and diffusion also depend on the presence of ST FAs and sterols, of which squalene is a precursor. In addition, squalene synthase is a putative branching point in the isoprenoid biosynthetic pathway, capable of directing carbon flux specifically to sterol biosynthesis, and therefore is considered a potential regulatory point of sterol metabolism (Devarenne et al., 2002). Based on the present results, this study demonstrated that salinity differently affects various components of quinoa seeds (Figure 6).

There are several possible reasons for the decrease in squalene content: (1) possible inhibition of squalene synthase; (2) redirection of the carbon flux from the biosynthesis of squalene to the biosynthesis of sterol; (3) a change in the ratio of NS/ST LCDs to preserve the fluidity and functionality of membranes; (4) a decrease in membrane permeability for ion movement as a protective reaction to an increase in the concentration of sodium ions; and (5) redirection of the NADPH cofactor to enhance the biosynthesis of proline in response to salinity, as both syntheses (squalene and proline) require NADPH.

## Conclusion

The new quinoa Q5 is a stress-tolerant line, with a high nutritional value and unique phytochemical composition. Despite some plant growth performance and grain quality decline, its outstanding nutritional value along with its biochemical traits makes it a suitable candidate for direct incorporation into the human diet, especially in resource-poor rural areas. It is generally agreed that the amino acid and FA composition of quinoa is the primary indicator determining the nutritional composition and illustrates its value as a food source. This quinoa line also presented a high content of squalene, which might be responsible for the positive results of the seeds’ bioactivities. Further work is needed to investigate the role of the environmental stress levels on the FA biosynthetic enzymes in different genotypes of quinoa for better understanding the mechanism by which FAs accumulate in the seeds. This work contributes to the growing discussion on alternative sustainable and healthier foods by presenting new information regarding this climate-resilient and salinity-adapted new line of quinoa. This information can potentially be used by the food and pharmaceutical industries in the development of new food and health products.

## Data Availability Statement

The raw data supporting the conclusions of this article will be made available by the authors, without undue reservation, to any qualified researcher.

## Author Contributions

KT: design of experimental trials, data collection and processing on plant growth performance, seed formation, seed traits analyzing, translation, writing, and editing of the manuscript. AM: seed chemistry lab analysis of minerals, squalene, fatty acids, data processing, and preparation of the first draft of the manuscript. BK: field and lab soil analysis, design of the manuscript, data processing on crop agronomic traits, and writing the first draft. AS: field and lab experiments, technical assistance on seed chemistry analysis of seed storage proteins, fatty acids, major minerals and squalene, and description of numerical data. AK: design of the manuscript and edition. KN: breeding and description of Q5 new genotypes and editing of the manuscript. ES: data analysis, statistical analysis, and editing of the manuscript. All authors contributed to the article and approved the submitted version.

## Conflict of Interest

The authors declare that the research was conducted in the absence of any commercial or financial relationships that could be construed as a potential conflict of interest.
